# Surgery for Colorectal Cancer: A Trigger for Liver Metastases Development? New Insights into the Underlying Mechanisms

**DOI:** 10.3390/biomedicines9020177

**Published:** 2021-02-11

**Authors:** Simran Grewal, Steven J. Oosterling, Marjolein van Egmond

**Affiliations:** 1 Department of Molecular Cell Biology and Immunology, Amsterdam University Medical Centers, 1007 MB Amsterdam, The Netherlands; m.vanegmond@amsterdamumc.nl; 2 Department of Surgery, Amsterdam University Medical Centers, 1081 HV Amsterdam, The Netherlands; 3 Cancer Center Amsterdam, Amsterdam University Medical Centers, 1081 HV Amsterdam, The Netherlands; 4 Department of Surgery, Spaarne Gasthuis, 2035 RC Haarlem, The Netherlands; SJ.oosterling@spaarnegasthuis.nl

**Keywords:** colorectal cancer, immunology, liver metastases, treatment

## Abstract

Surgery is a crucial intervention and provides the best chance of cure for patients with colorectal cancer. Experimental and clinical evidence, however, suggests that paradoxically surgery itself may precipitate or accelerate tumor recurrence and/or liver metastasis development. This review addresses the various aspects of surgery-induced metastasis formation and sheds light on the role of inflammation as potential trigger for metastasis development. Understanding these mechanisms may provide potential new perioperative interventions to improve treatment outcomes, and as such could transform the perioperative timeframe from a facilitator of metastatic progression to a window of opportunity to reduce the risk of liver metastasis development. Ultimately, this can potentially improve long-term survival rates and quality of life in patients with colorectal cancer.

## 1. Introduction

Colorectal cancer (CRC) is the third most common cancer subtype worldwide with over one million new cases diagnosed in 2018 [1]. Metastatic disease represents the primary cause of mortality in CRC. Liver metastasis is the most common site of distant spread, accounting for approximately 15–25% of CRC patients at the time of primary diagnosis. Another 18–25% patients will develop distant metastases within 5 years from the first diagnosis [2]. Indications for treatment of CRC liver metastases with curative intent have expanded rapidly. The treatment regime for most patients with CRC consists of surgical resection, either with or without chemo-and/or radiotherapy [3]. Prognosis and the need for adjuvant therapy is primary based on the tumor-node-metastasis (TNM)-stage, which describes the extent of invasion of the primary tumor (T), lymph nodes (N) and distant metastases (M). The five-year survival rate for stages I-III CRC is up to 80%. For stage IV CRC, which represents 20% of all cases, it is only about 13% [4].

Although surgical excision of primary tumor can save or extend life, it has been hypothesized that surgery itself may precipitate or accelerate tumor recurrence and/or liver metastasis development, as surgery may generate a permissive environment for tumor growth [5,6]. This is likely not specific for CRC surgery, as effects of surgery on the metastatic process have also been observed in patients with breast cancer. Among women who were operated for breast cancer, 30% of node-negative and 75% of node-positive women still developed distant metastasis [7].

Therefore, addressing the mechanisms involved in the pro-tumorigenic perioperative period may provide insight into new therapeutic strategies to improve cancer outcomes.

**Figure 1:**
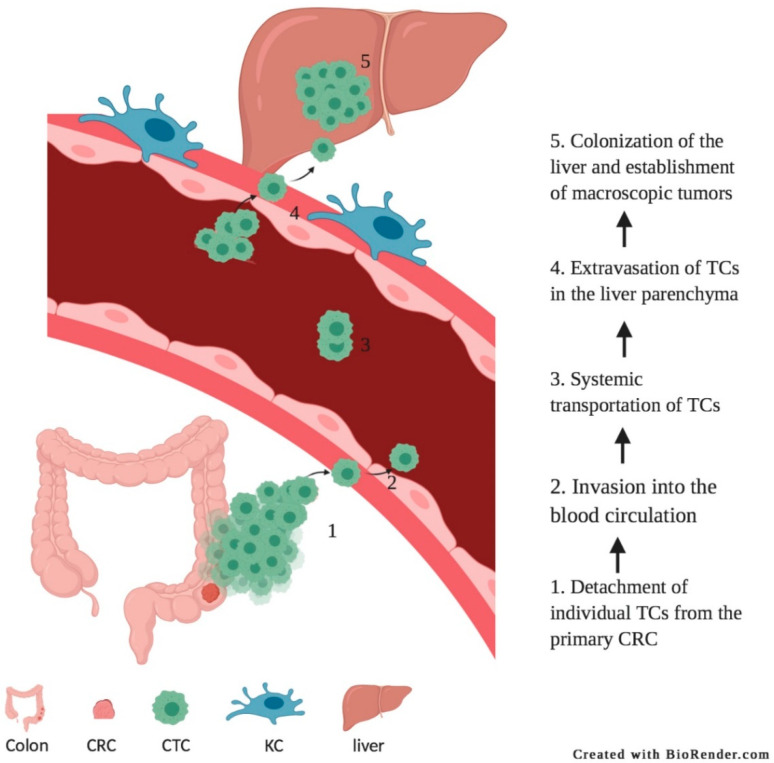
Classical route of liver metastases development. Detachment of individual tumor cells from the primary tumor (step 1), intravasation of tumor cells into the circulatory system (step 2), systemic transportation of tumor cells (step 3), extravasation of tumor cells into parenchyma of distant tissue (step 4), colonization of distant organs and establishment of macroscopic tumors (step 5). TC = tumor cell.

During classical metastasis development, tumor cells undergo a complex cascade of events to form metastases in target organs [8,9] (Figure 1). First, cancer cells need to “*escape from the primary tumor*” and must become motile and invasive. This requires changes in cell–cell and cell–extracellular matrix (ECM) contacts. The new blood vessels that develop in the primary tumor during growth, defined as angiogenesis, provide an escape route whereby tumor cells can enter into the vascular system (*intravasation*). Next, tumor cells need to “*survive in the circulation*”. Binding to and covering themselves with platelets has shown to protect circulating tumor cells (CTCs) from shear stress, immune cells and host defense mechanisms [10]. Cancer cells need to “*arrest at a new site*”, which is followed by “*extravasation into the tissue*”. Once the tumor cells have reached the new site, cells need to grow out and form micrometastases and finally macroscopic metastases. Each stage of metastasis imposes different, often harsh conditions and challenges for cancer cells. As the cascade progresses, the number of viable cancer cells which survive and successfully complete each stage decreases. Thus, classical metastasis is a complex and inefficient process because of the multiplicity of events that are required or need to be overcome.

In this review, we summarize the growing evidence that supports the concept that surgery for primary colorectal cancer can actually increase the risk of new liver metastasis development. In addition, we will review the perioperative factors that may enhance postoperative tumor growth and the therapeutic implications that might be useful in counteracting this phenomenon.

## 2. Adhesion of Circulating Tumor Cells

It has been demonstrated that circulating tumor cells (CTCs) are present in peripheral blood and portal circulation of patients with CRC pre-operatively [11,12,13,14]. Moreover, it was shown that the number of CTCs increased during, or shortly after resection in both peripheral and portal blood circulation, which suggest that manipulation of the primary CRC can lead to dissemination of tumor cells [15,16,17]. It has been proposed that these CTCs have a high prognostic value independently of already established criteria such as tumor staging [18,19]. Patients with CRC harboring >5 CTC per 7.5 mL blood had a worse prognosis with an eight-times higher risk of developing metastasis within a year [11]. A recent meta-analysis summarized 20 relevant studies including 3687 patients and provided strong evidence for peripheral blood CTCs predicting poor disease progression and survival in patients with non-metastatic CRC [20]. However, as implantation of CTCs is a highly inefficient process, spillage of tumor cells during surgery likely cannot fully explain the high incidence of metastases development.

In order to form a metastasis, CTCs first need to adhere in the target organ. Adhesion molecules are expressed on cancer cells and cells of the target organ and play a crucial role in metastatic progression [21]. Adhesion molecules generate the initial cell-cell contacts that lead to cancer extravasation and subsequent organ colonization. Expression of several adhesion molecules, such as E-selectin, vascular cellular adhesion molecule (VCAM)-1 and ICAM-1, was increased in the liver during metastatic invasion [22]. Selectins are vascular cell adhesion molecules involved in adhesive interactions of leucocytes and platelets and endothelium within the blood circulation. Platelets promote tumorigenesis and metastasis via a number of complementary mechanisms, including (a) aggregation around the CTCs to form a platelet ‘cloak’ thus shielding them from high shear forces generated by blood flow, lodging them into the vessel wall [23,24]. Additionally, platelets protect tumor cells from attack by the immune system [25], and release permeability factors and degradative enzymes that assist tumor cell extravasation from the circulation [26]. The release of growth and angiogenic factors also help to facilitate the establishment of metastasis [27]. Selectins are also present within the liver sinusoids, regulating CRC cell arrest and extravasation in the liver [28]. Inhibition or downregulation of selectin expression resulted in attenuation of experimental liver metastasis [28]. In contrast, overexpression of selectins in the liver redirected metastasis to this organ, thereby confirming the role of selectins in this process [29]. Furthermore, it has been reported that tumor cell derived factors up-regulate the synthesis of cytokine release, like (TNF)α, IL-1 and IL-6, by immune cells [30,31]. These cytokines were shown to enhance the expression of E-selectin on the surface of endothelial cells -including the liver sinusoidal endothelium-, which was suggested to facilitate the tumor cells adhesion and enable metastases outgrowth. Moreover, enhanced tumor cell adhesion by surgery-released cytokines was demonstrated both in vivo and in vitro [32,33,34].

Integrins are transmembrane glycoproteins consisting of α and β subunits heterodimers [35]. They mediate cell adhesion and directly bind to ECM components such as fibronectin and collagen [36]. Altered integrin expression patterns have been linked to many types of cancer [37,38,39]. Integrins contribute to the metastatic cascade by upregulating the expression of matrix metalloproteinase genes and facilitating protease activation and function of the ECM interface [40]. Interaction between integrin heterodimers and ECM proteins of the target organ initiates tumor cell attachment in the liver sinusoids, which eventually lead to cancer cell extravasation and subsequent organ colonization [41].

Surgical trauma, which is inevitable during resection of primary tumor, initiates systemic inflammation and leads to rapid activation of innate immune cells. These cells are potent producers of inflammatory mediators like reactive oxygen species (ROS) [42]. It was demonstrated that incubation of mesothelial cells with ROS enhanced expression of the adhesion molecules ICAM-1 and VCAM-1 [43]. This consequently increased tumor cell adherence to mesothelial cells. Additionally, impairment of the mesothelial monolayer of the peritoneal wall and liver micro-vasculature after abdominal surgery was demonstrated [44,45]. This resulted in formation of intercellular gaps and exposure of extracellular matrix (ECM) proteins, which served as preferable adhesion sites for tumor cells. Incubation of tumor cells with antibodies against integrin subunits prevented surgery-induced tumor cell adhesion and tumor outgrowth in the peritoneum or the liver [45,46]. In vivo models suggested that interactions between integrins on the tumor cell surface and exposed ECM played a major role in metastases development after surgery. Thus, surgery creates permissive circumstances for tumor cells to adhere to adhesion molecules in ECM of target organs and thereby increase chances of metastases development.

## 3. Anastomotic Leakage and Bacterial Translocation

Approximately, 8–10% of patients encounter an anastomotic leakage after resection of the colorectal tumor [47,48]. Anastomotic leakage is often defined as ‘a defect in the bowel wall at the anastomotic site, leading to communication of intra-and extraluminal compartments’ [49]. The relationship between anastomotic leakage and oncological outcome is a controversial issue. Although several studies have shown a relationship between anastomotic leakage and disease recurrence as well as overall survival [50,51,52,53], other studies have not found an adverse effect on oncological outcome [54,55]. Nonetheless, recent meta-analyses showed increased local recurrence and reduced long-term survival from colorectal cancer following anastomotic leakage [56,57,58,59].

Colorectal surgery has been shown to lead to bacterial translocation into the circulation and abdominal cavity [60,61,62,63,64]. LPS has been detected in post-surgical plasma of patients [65,66] and elevation of LPS concentration in blood was accompanied by intestinal permeability, which suggest that the gut epithelial barrier is impaired post-operatively [67]. In line with this, we have shown in an animal model that injection of LPS in the peritoneal cavity increased tumor cell adhesion in the liver, which may contribute to a poor oncological outcome in patients with anastomotic leakage [44].

## 4. Surgery Induced Activation of Immune Cells

Bacterial components are potent triggers of inflammatory immune response by acting on Toll-like receptors (TLRs) [68]. LPS is the main ligand for TLR4, which is expressed by a wide variety type of immune cells [69]. As bacterial components are released during surgery, Kupffer cells (KCs) and polymorphonuclear cells (PMNs) expressing TLRs that recognize the bacterial products, become activated. This results in release of high levels of cytokines and ROS production [44,70]. ROS enhanced expression of adhesion molecules ICAM-1 and VCAM-1 of mesothelial cells [43]. Furthermore, exposing endothelial cell layers to ROS led to detachment and subtraction of endothelial cells and subsequently to enhanced adhesion of tumor cells to exposed underlying extracellular matrix proteins in in vitro assays [44,71]. In vivo, ROS production has a damaging effect on the integrity of the liver vasculature, resulting in exposure of sub-cellular extracellular matrix (ECM) components to which circulating tumor cells can adhere in an experimental rat model [46].

The immune system may be an important factor in determining the outcome of people with cancer [72]. A scoring system has been developed to classify tumors based on the quantification of CD3 and CD8 cells [73]. Two major immune groups have been described, referred to as: ‘inflamed or hot’ tumors and ‘cold’ tumors. An ‘inflamed’ tumor is highly infiltrated with T-cells and has a strong pre-existing adaptive immune system. In the ‘cold’ tumors, T cells are either found at the edge of tumor sites without being able to infiltrate them (excluded phenotype), or absent (desert phenotype). The excluded phenotype likely reflects the intrinsic inability of the host immune system to effectively mount a T-cell mediated immune response and the ability of the tumor to escape such response by hindering T cell infiltration. In contrast, in immunologically hot tumors the immune system can trigger an anti-tumor response. Therefore, this may contribute to a better oncologic outcome of patients with a ‘hot’ tumor as compared to patients with a ‘cold’ tumor.

The presence of more intra-tumoral macrophages has been associated with a favorable disease-free survival in CRC [74]. Nonetheless, some studies did not find an association between the number of macrophages and patient survival, so consensus has not yet been reached [75,76,77]. Macrophages are phagocytic cells with pro- or anti-inflammatory properties, depending on their phenotype [78,79,80]. The role of macrophages in tumor progression acts as a double-edged sword, since they can both promote tumor elimination as well as stimulate tumor growth [81]. Inflammatory macrophages (also referred to as M1-like or classically activated macrophages) are involved in the inflammatory response, pathogen clearance and anti-tumor immunity. Conversely, immune modulatory macrophages (also referred to as M2-like or alternatively-activated macrophages) are associated with an immune suppressive responses, wound healing and pro-tumorigenic properties, including induction of angiogenesis and facilitation of metastases.

A post-operative imbalance in pro-and anti-inflammatory immune responses may debilitate antitumor cytotoxicity, or promote immunomodulatory and wound healing functions, thereby facilitating metastasis outgrowth. The per- and postoperative acute phase responses initiate systemic inflammation leading to rapid activation of innate immune cells and subsequently to increased cytokines production [30,82]. The cytokines which play a dominant role in distant metastasis formation are tumor necrosis factor (TNF)α, IL-1 and IL-6, which are released per-and post-operatively [31,83]. These cytokines can, by enhancing expression of adhesion molecules, increase adhesion of tumor cells in vivo and in vitro [33,34]. Adhesion molecules like ICAM-1, VCAM-1 and E-selectin on endothelial cells are activated and their increased expression has also been shown to promote adhesion of tumor cells in vivo and in vitro [33,34].

To counterbalance the effects of the acute phase response, compensatory anti-inflammatory mediators are released [84]. It was demonstrated in immunodeficient mice and patients undergoing immunosuppressive therapies that an unbalanced systemic compensatory response to acute phase responses may result in immune suppression and hereby render the patient susceptible for post-operative infections and hampered anti-tumor immunity [85].

**Figure 2:**
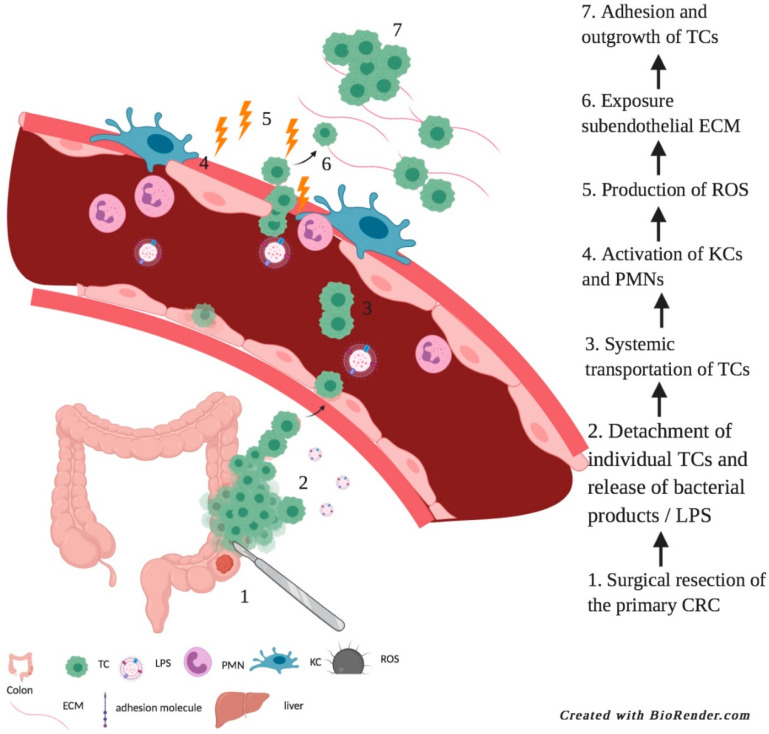
Surgery-induced liver metastases. Surgical resection of the primary CRC (step 1) causes release of bacterial products (LPS) and tumor cells (step 2). Systemic transportation of TCs (3) and activation of KCs and PMNs (4) which produce ROS (5). This leads to exposure of sub-endothelial ECM (6). Disseminated tumor cells adhere to the exposed ECM through and develop into liver metastases (7). TC = tumor cell; KC = Kupffer cell; PMN = polymorphonuclear cell; ROS; reactive oxygen species; ECM = extracellular matrix.

In conclusion, ‘surgery-induced liver metastasis development’ represents an additional route of metastasis formation that is initiated per- and post-operatively and which short-cuts several of the steps of the classical metastasis model. Handling of the tumor during surgery may result in shedding of tumor cells. Moreover, we have demonstrated in animal models that surgical abdominal trauma, and exposure to microbial factors led to impairment of liver vasculature, subsequently causing the exposure of the sub-endothelial extracellular matrix (ECM) proteins to which tumor cells adhere through their adhesion molecules and grow out into metastasis formation (Figure 2).

## 5. Potential Perioperative Interventions to Prevent Metastases Development

Most cancer patients with CRC will not die because of their primary tumor, which can be removed by surgery, but rather as a result of cancer metastasis. Therefore, the biggest challenge is not the removal of the primary tumor, but the effective prevention of metastasis development. As minimally invasive surgical resection remains the cornerstone of CRC treatment, the perioperative window simultaneously removes the bulk of the tumor and provides an opportunity to counteract adhesion of CTCs, hereby reducing the risk of recurrence and/or liver metastasis development.

## 6. Limiting Surgical Trauma?

We previously demonstrated in our animal model that enhanced numbers of adhered tumor cells were present in the livers of rats that underwent a colectomy procedure, compared to the livers of control rats or rats from the laparotomy group (sham operation) [61]. Furthermore, animals from the colectomy group developed significantly more liver metastases compared to the sham and control group. These results suggest that the extent of surgical trauma may enhance increased locoregional and distant tumor development after primary CRC surgery, supporting the proposition that reducing tissue damage may lead to better patient prognosis.

Laparoscopic surgery is nowadays considered as preferable technique for curative treatment of patients with CRC. Instead of operating through one large abdominal incision, several small incisions are made, and surgery is performed with smaller instruments. Several studies have demonstrated that laparoscopic surgery results in less perioperative blood loss, faster postoperative recovery, less pain and a shorter length of hospital stay [86].

Whether laparoscopy has benefits for oncological outcome is less clear. One of the early studies on laparoscopy-assisted colectomy in CRC was conducted by Lacy and co-workers in which they compared the efficacy of laparoscopic-assisted colectomy and open colectomy for the treatment of non-metastatic colon cancer in terms of tumor recurrence and survival. A total of 219 patients were included in this randomized controlled trial. In addition to the abovementioned beneficial effects of the procedure, also overall survival was higher in patients undergoing laparoscopy-assisted colectomy compared to open surgery [87]. However, this study is controversial, as these promising results could only be reproduced in a subgroup of patients in two recent large trials, i.e., the Barcelona trial and Colorectal Cancer Laparoscopic or Open Resection (COLOR) trials. The Barcelona trial randomized 105 patients to laparoscopic assisted colectomy and 101 patients to open colectomy. The most significant finding of the trial was the cancer related mortality, which was significantly lower in the laparoscopic assisted group (95) compared to the open surgery group (21%), *p* = 0.03. However, this difference in cancer related survival was due to outcomes of a subgroup of patients with stage III disease. In the COLOR II trial, patients were randomly assigned to undergo either laparoscopic resection of rectal cancer or an open procedure. Long-term rates of locoregional recurrence at 3 years after surgery was 5% for both laparoscopic and open surgery groups [88]. However, laparoscopic surgery in patients with cancer in the lower third of the rectum was associated with a lower locoregional recurrence rate than for open surgery. The disease-free survival rates at 3 years for the overall groups were 74.8% after laparoscopic surgery and 70.8% after open surgery, which was not significantly different. In the Conventional versus Laparoscopic Assisted Surgery in Colorectal Cancer (CLASICC) trial, a randomized study to determine the effect of laparoscopic surgery on rectal-cancer outcomes involving 381 patients, the locoregional recurrence rate at 3 years was 9.7% after laparoscopic surgery versus 10.1% after open surgery [89,90,91]. Thus, possibly with the exception of specific subgroups, laparoscopic colectomy is overall not superior to open approach with respect to oncological outcome of cancer patients.

## 7. Blocking Tumor Cell Adhesion

Clinical and experimental evidence shows the important role of adhesion molecules in liver metastases formation. Several studies suggested that modulation of endothelial adhesion molecules expression may provide a useful strategy to prevent liver metastases. Targeting E-selectin by antibodies was shown to decrease tumor cell adhesion and reduce liver metastases development [92]. Furthermore, inhibition of VCAM-1 expression by antibody-mediated blockade of IL-1, TNFα and IL-18 also impaired the retention of cancer cells in the liver sinusoids and reduced liver metastases development [93]. We previously demonstrated that surgery led to endothelial vascular damage in the liver with exposure of sub-endothelial ECM to which tumor cells adhered through α2β1 integrins. In animal models, development of metastases was significantly diminished after functional blockade of integrin α2 on colon carcinoma cells [46]. Therefore, inhibiting tumor cell adhesion might be a promising approach to prevent surgery-induced liver metastases. However, it is important to keep in mind that surgery unavoidably introduces tissue injury. Immediately after surgery wound healing processes are initiated, in which the interaction of immune cells with endothelial cells or ECM proteins play an essential role. Integrins, and other adhesion molecules play a central role in wound healing, that may be disrupted by blocking them [94,95]. For instance, it was demonstrated that integrin β1 is involved in keratinocyte migration during cutaneous repair. Hampered wound healing after colon resection may have serious consequences for the bowel anastomosis as leakage and potential sepsis have been associated with poor long-term oncological outcome [52,96,97]. As such, great care must be taken to not to interfere with wound healing processes. Although blocking adhesion molecules diminishes tumor cell adhesion and subsequently may decrease metastases outgrowth postoperatively, it may also lead to severe side effects in surgical patients, which potentially countermands any beneficial effects of the therapy. Therefore, caution should be taken in attempting to treat patients with adhesion molecule blocking agents to improve patient’s outcome.

## 8. Reducing Inflammatory Responses

Surgery induced immune responses result in activation of immune cells, which can lead to production of high concentrations of ROS [98]. This subsequently may damage endothelial lining and results in exposure of the sub-endothelial ECM. As this is a favorable adhesion site for tumor cells, reducing ROS production could prevent metastases development. Although treatment with ROS scavengers initially led to decreased tumor cell adhesion in animal models, it paradoxically promoted liver metastases outgrowth [44]. This was likely due to the role of ROS in tumor cell killing by macrophages [99]. As such, ROS has dual and conflicting actions. Developing ROS scavengers with short half-life, which may interrupt early ROS production during surgery, hereby leading to less damaged liver vasculature and tumor cells adherence, while preserving long-term macrophage function may potentially have therapeutic potential [44]. A few randomized controlled trials and retrospective studies studied the impact of perioperative treatment with COX inhibitors, which block catecholamines and/or prostaglandins. Although some moderate effects on survival were suggested, overall results are inconclusive [100,101].

## 9. Oral Antibiotics for Decontamination of the Digestive Tract

The microbiome is the microbial ecosystem of the body and resides largely in the digestive tract. In recent years, DNA and RNA sequencing studies have revealed that the diversity and metabolic interactions of this microbial community greatly influence the development of infection and disease [102]. When balance in the microbiome is lost and potentially pathogenic microorganisms predominate the bowel environment, a ‘disease-promoting microbiome’ occurs that facilitates the occurrence of disease and infectious complications [103]. Despite improvements in surgical equipment and techniques, patients undergoing colorectal surgery are at risk for development of infectious complications, such as surgical site infection (SSI) and anastomotic leakage (AL) [104,105]. Several studies have also linked AL with increased tumor recurrence and higher cancer-specific mortality [50,51,52].

Selective Decontamination of the Digestive Tract (SDD) is based on the administration of oral non-absorbable antibiotics and fungicides to eliminate potentially pathogenic microorganisms in the bowel [106]. SDD minimizes the impact of infections by potentially pathogenic microorganisms that are endogenous to the patient. These microorganisms colonize the digestive tract and consist predominantly of aerobic Gram-negative bacteria, Staphylococcus aureus and fungi, against which SDD is effective. SDD decreases infectious complications in oesophagogastric cancer surgery [107]. In 2009 Roos and co-workers published a retrospective case–control study and a smaller single-centre RCT of SDD in patients undergoing gastrointestinal surgery [108,109]. Their RCT demonstrated a significant decrease in infectious complications and anastomotic leak rates as a combined endpoint in patients undergoing various gastrointestinal operations, including colorectal, oesophageal and gastric resections for both benign and malignant disease [109]. Recently, we published the results of the SELECT trial in which the effect of SDD on surgical site infection and anastomotic leakage was evaluated in a multicenter RCT. SDD reduced infectious complications after colorectal cancer resection but did not significantly reduce anastomotic leakage. However, the trial had to be stopped after interim analysis demonstrated that superiority was no longer attainable. In a systematic review and meta-analysis (Grewal et al., submitted), the impact of MBP and OAB on SSI and AL was assessed in patients undergoing elective surgical resection of colorectal cancer. Preoperative OAB prophylaxis, in combination with MBP and standard intravenous antibiotic prophylaxis, was associated with a significant reduction in rates of SSI and AL. Interestingly, OAB as SDD seems to be more effective compared to non-selective antibiotics in reducing the risk of surgical site infection and anastomotic after colorectal cancer surgery.

**Figure 3:**
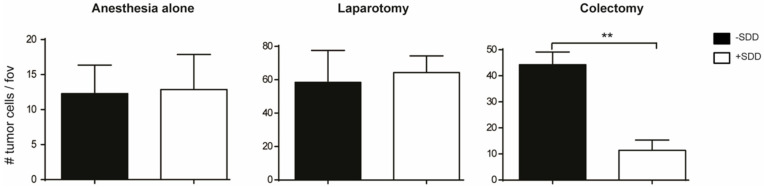
Numbers of tumor cells per field of view (fov) in the liver of rats that were treated without (black bars) or with SDD (white bars) regime and underwent anesthesia alone, laparotomy or colectomy. ** *p* < 0.01.

We have previously demonstrated in an experimental model that abdominal surgery lead to bacterial translocation and induction of an inflammatory response. We observed significantly enhanced distant adherence of tumor cells in the liver of rats and subsequent tumor outgrowth [61]. Interestingly, the enhanced tumor cell adherence was prevented by pre-operative SDD (Figure 3).

Our hypothesis is that surgical trauma leads to activation of the immune system and damage of endothelial lining of liver vasculature by release of reactive oxygen species (ROS). Sub-endothelial extracellular matrix molecules are exposed by ROS, facilitating the adhesion of circulating tumor cells to the exposed extracellular matrix molecules and outgrowth of liver metastases. Bacterial components are known to be potent triggers for induction of inflammatory response, therefore bacterial contamination may escalate the above described mechanism. Bacterial components are recognized by Toll-like receptors (TLR) and induce activation of immune cells. Consequently, release of ROS by activated immune cells may facilitate the adherence of tumor cells. By pre-operatively elimination of gram-negative bacteria in the colon, we anticipate that surgery-induced pro-tumorigenic immune responses may be avoided to improve oncological outcome.

## 10. Concluding Remarks

Liver metastasis is a common cause of morbidity and mortality in colorectal cancer patients. Both experimental and clinical evidence support the notion that surgery, which is intended as a curative option by removing the tumor mass, can paradoxically also augment development of metastases. In this review we have highlighted the role of bacterial products and inflammatory responses to surgical trauma, which may underlie many aspects of poor oncological outcome. The perioperative period provides an “window of opportunity” for developing relevant therapies to reduce the risk of metastasis development. We propose that preoperative use of SDD to reduce the load of gut bacteria, may subsequently decrease harmful inflammatory responses with concomitant damage to liver sinusoids, increased adhesion of CTCs and development of metastases.

## Data Availability

Data is contained within the article. The data presented in this study are available in this article (Figure 3).

## References

[1] Bray F., Ferlay J., Soerjomataram I., Siegel R.L., Torre L.A., Jemal A. (2018). Global cancer statistics 2018: GLOBOCAN estimates of incidence and mortality worldwide for 36 cancers in 185 countries. CA A Cancer J. Clin..

[2] Kow A.W.C. (2019). Hepatic metastasis from colorectal cancer. J. Gastrointest. Oncol..

[3] Matsuda T., Yamashita K., Hasegawa H., Oshikiri T., Hosono M., Higashino N., Yamamoto M., Matsuda Y., Kanaji S., Nakamura T. (2018). Recent updates in the surgical treatment of colorectal cancer. Ann. Gastroenterol. Surg..

[4] Siegel R.L., Miller K.D., Goding Sauer A., Fedewa S.A., Butterly L.F., Anderson J.C., Cercek A., Smith R.A., Jemal A. (2020). Colorectal cancer statistics, 2020. CA A Cancer J. Clin..

[5] Paget S. (1989). The distribution of secondary growths in cancer of the breast. 1889. Cancer Metastasis Rev..

[6] Tohme S., Simmons R.L., Tsung A. (2017). Surgery for Cancer: A Trigger for Metastases. Cancer Res..

[7] Demicheli R., Dillekås H., Straume O., Biganzoli E. (2019). Distant metastasis dynamics following subsequent surgeries after primary breast cancer removal. Breast Cancer Res. BCR.

[8] Hapach L.A., Mosier J.A., Wang W., Reinhart-King C.A. (2019). Engineered models to parse apart the metastatic cascade. NPJ Precis. Oncol..

[9] Jin K., Gao W., Lu Y., Lan H., Teng L., Cao F. (2012). Mechanisms regulating colorectal cancer cell metastasis into liver (Review). Oncol. Lett..

[10] Weidle U.H., Birzele F., Krüger A. (2015). Molecular targets and pathways involved in liver metastasis of colorectal cancer. Clin. Exp. Metastasis.

[11] Tsai W.S., Chen J.S., Shao H.J., Wu J.C., Lai J.M., Lu S.H., Hung T.F., Chiu Y.C., You J.F., Hsieh P.S. (2016). Circulating Tumor Cell Count Correlates with Colorectal Neoplasm Progression and Is a Prognostic Marker for Distant Metastasis in Non-Metastatic Patients. Sci. Rep..

[12] Arrazubi V., Mata E., Antelo M.L., Tarifa A., Herrera J., Zazpe C., Teijeira L., Viudez A., Suárez J., Hernández I. (2019). Circulating Tumor Cells in Patients Undergoing Resection of Colorectal Cancer Liver Metastases. Clinical Utility for Long-Term Outcome: A Prospective Trial. Ann. Surg. Oncol..

[13] Bork U., Grützmann R., Rahbari N.N., Schölch S., Distler M., Reissfelder C., Koch M., Weitz J. (2014). Prognostic relevance of minimal residual disease in colorectal cancer. World J. Gastroenterol..

[14] Bork U., Rahbari N.N., Schölch S., Reissfelder C., Kahlert C., Büchler M.W., Weitz J., Koch M. (2015). Circulating tumour cells and outcome in non-metastatic colorectal cancer: A prospective study. Br. J. Cancer.

[15] Conzelmann M., Linnemann U., Berger M.R. (2005). Detection of disseminated tumour cells in the liver of colorectal cancer patients. Eur. J. Surg. Oncol..

[16] Yamaguchi K., Takagi Y., Aoki S., Futamura M., Saji S. (2000). Significant detection of circulating cancer cells in the blood by reverse transcriptase-polymerase chain reaction during colorectal cancer resection. Ann. Surg..

[17] Martin O.A., Anderson R.L., Narayan K., MacManus M.P. (2017). Does the mobilization of circulating tumour cells during cancer therapy cause metastasis?. Nat. Rev. Clin. Oncol..

[18] Seeberg L.T., Brunborg C., Waage A., Hugenschmidt H., Renolen A., Stav I., Bjornbeth B.A., Borgen E., Naume B., Brudvik K.W. (2017). Survival Impact of Primary Tumor Lymph Node Status and Circulating Tumor Cells in Patients with Colorectal Liver Metastases. Ann. Surg. Oncol..

[19] Huang X., Gao P., Song Y., Sun J., Chen X., Zhao J., Xu H., Wang Z. (2015). Meta-analysis of the prognostic value of circulating tumor cells detected with the CellSearch System in colorectal cancer. BMC Cancer.

[20] Lu Y.J., Wang P., Peng J., Wang X., Zhu Y.W., Shen N. (2017). Meta-analysis Reveals the Prognostic Value of Circulating Tumour Cells Detected in the Peripheral Blood in Patients with Non-Metastatic Colorectal Cancer. Sci. Rep..

[21] Stasinopoulos I., Penet M.-F., Krishnamachary B., Bhujwalla Z.M. (2010). Molecular and functional imaging of invasion and metastasis: Windows into the metastatic cascade. Cancer Biomark..

[22] Khatib A.M., Auguste P., Fallavollita L., Wang N., Samani A., Kontogiannea M., Meterissian S., Brodt P. (2005). Characterization of the host proinflammatory response to tumor cells during the initial stages of liver metastasis. Am. J. Pathol..

[23] McCarty O.J.T., Jadhav S., Burdick M.M., Bell W.R., Konstantopoulos K. (2002). Fluid shear regulates the kinetics and molecular mechanisms of activation-dependent platelet binding to colon carcinoma cells. Biophys. J..

[24] Burdick M.M., Konstantopoulos K. (2004). Platelet-induced enhancement of LS174T colon carcinoma and THP-1 monocytoid cell adhesion to vascular endothelium under flow. Am. J. Physiol. Cell Physiol..

[25] Nieswandt B., Hafner M., Echtenacher B., Männel D.N. (1999). Lysis of tumor cells by natural killer cells in mice is impeded by platelets. Cancer Res..

[26] Deryugina E.I., Quigley J.P. (2006). Matrix metalloproteinases and tumor metastasis. Cancer Metastasis Rev..

[27] Pinedo H.M., Verheul H.M., D’Amato R.J., Folkman J. (1998). Involvement of platelets in tumour angiogenesis?. Lancet.

[28] Gout S., Tremblay P.L., Huot J. (2008). Selectins and selectin ligands in extravasation of cancer cells and organ selectivity of metastasis. Clin. Exp. Metastasis.

[29] Läubli H., Borsig L. (2010). Selectins as mediators of lung metastasis. Cancer Microenviron. Off. J. Int. Cancer Microenviron. Soc..

[30] Eichbaum C., Meyer A.S., Wang N., Bischofs E., Steinborn A., Bruckner T., Brodt P., Sohn C., Eichbaum M.H. (2011). Breast cancer cell-derived cytokines, macrophages and cell adhesion: Implications for metastasis. Anticancer Res..

[31] Veenhof A.A., Vlug M.S., van der Pas M.H., Sietses C., van der Peet D.L., de Lange-de Klerk E.S., Bonjer H.J., Bemelman W.A., Cuesta M.A. (2012). Surgical stress response and postoperative immune function after laparoscopy or open surgery with fast track or standard perioperative care: A randomized trial. Ann. Surg..

[32] Sturm J.W., Magdeburg R., Berger K., Petruch B., Samel S., Bonninghoff R., Keese M., Hafner M., Post S. (2003). Influence of TNFA on the formation of liver metastases in a syngenic mouse model. Int. J. Cancer.

[33] Ten Kate M., Hofland L.J., van Grevenstein W.M., van Koetsveld P.V., Jeekel J., van Eijck C.H. (2004). Influence of proinflammatory cytokines on the adhesion of human colon carcinoma cells to lung microvascular endothelium. Int. J. Cancer.

[34] Ziprin P., Ridgway P.F., Pfistermuller K.L., Peck D.H., Darzi A.W. (2003). ICAM-1 mediated tumor-mesothelial cell adhesion is modulated by IL-6 and TNF-alpha: A potential mechanism by which surgical trauma increases peritoneal metastases. Cell Commun. Adhes..

[35] Barczyk M., Carracedo S., Gullberg D. (2010). Integrins. Cell Tissue Res..

[36] Desgrosellier J.S., Cheresh D.A. (2010). Integrins in cancer: Biological implications and therapeutic opportunities. Nat. Rev. Cancer.

[37] Seguin L., Desgrosellier J.S., Weis S.M., Cheresh D.A. (2015). Integrins and cancer: Regulators of cancer stemness, metastasis, and drug resistance. Trends Cell Biol..

[38] Hamidi H., Pietila M., Ivaska J. (2016). The complexity of integrins in cancer and new scopes for therapeutic targeting. Br. J. Cancer.

[39] Raab-Westphal S., Marshall J.F., Goodman S.L. (2017). Integrins as Therapeutic Targets: Successes and Cancers. Cancers.

[40] Munshi H.G., Stack M.S. (2006). Reciprocal interactions between adhesion receptor signaling and MMP regulation. Cancer Metastasis Rev..

[41] Cox D., Brennan M., Moran N. (2010). Integrins as therapeutic targets: Lessons and opportunities. Nat. Rev. Drug Discov..

[42] Van der Bij G.J., Oosterling S.J., Beelen R.H., Meijer S., Coffey J.C., van Egmond M. (2009). The perioperative period is an underutilized window of therapeutic opportunity in patients with colorectal cancer. Ann. Surg..

[43] Van Grevenstein W.M., Aalbers A.G., Ten Raa S., Sluiter W., Hofland L.J., Jeekel H., van Eijck C.H. (2007). Surgery-derived reactive oxygen species produced by polymorphonuclear leukocytes promote tumor recurrence: Studies in an in vitro model. J. Surg. Res..

[44] Gul N., Bogels M., Grewal S., van der Meer A.J., Rojas L.B., Fluitsma D.M., van den Tol M.P., Hoeben K.A., van Marle J., de Vries H.E. (2011). Surgery-induced reactive oxygen species enhance colon carcinoma cell binding by disrupting the liver endothelial cell lining. Gut.

[45] Oosterling S.J., van der Bij G.J., Bogels M., ten Raa S., Post J.A., Meijer G.A., Beelen R.H., van Egmond M. (2008). Anti-beta1 integrin antibody reduces surgery-induced adhesion of colon carcinoma cells to traumatized peritoneal surfaces. Ann. Surg..

[46] van der Bij G.J., Oosterling S.J., Bogels M., Bhoelan F., Fluitsma D.M., Beelen R.H., Meijer S., van Egmond M. (2008). Blocking alpha2 integrins on rat CC531s colon carcinoma cells prevents operation-induced augmentation of liver metastases outgrowth. Hepatology.

[47] Choi H.K., Law W.L., Ho J.W. (2006). Leakage after resection and intraperitoneal anastomosis for colorectal malignancy: Analysis of risk factors. Dis. Colon Rectum.

[48] Frasson M., Flor-Lorente B., Rodriguez J.L., Granero-Castro P., Hervas D., Alvarez Rico M.A., Brao M.J., Sanchez Gonzalez J.M., Garcia-Granero E. (2015). Risk Factors for Anastomotic Leak After Colon Resection for Cancer: Multivariate Analysis and Nomogram From a Multicentric, Prospective, National Study With 3193 Patients. Ann. Surg..

[49] Van Rooijen S.J., Jongen A.C., Wu Z.-Q., Ji J.-F., Slooter G.D., Roumen R.M., Bouvy N.D. (2017). Definition of colorectal anastomotic leakage: A consensus survey among Dutch and Chinese colorectal surgeons. World J. Gastroenterol..

[50] Belt E.J., Stockmann H.B., Abis G.S., de Boer J.M., de Lange-de Klerk E.S., van Egmond M., Meijer G.A., Oosterling S.J. (2012). Peri-operative bowel perforation in early stage colon cancer is associated with an adverse oncological outcome. J. Gastrointest. Surg. Off. J. Soc. Surg. Aliment. Tract.

[51] Kube R., Mroczkowski P., Granowski D., Benedix F., Sahm M., Schmidt U., Gastinger I., Lippert H. (2010). Anastomotic leakage after colon cancer surgery: A predictor of significant morbidity and hospital mortality, and diminished tumour-free survival. Eur. J. Surg. Oncol..

[52] Law W.L., Choi H.K., Lee Y.M., Ho J.W., Seto C.L. (2007). Anastomotic leakage is associated with poor long-term outcome in patients after curative colorectal resection for malignancy. J. Gastrointest. Surg..

[53] Goto S., Hasegawa S., Hida K., Uozumi R., Kanemitsu Y., Watanabe T., Sugihara K., Sakai Y. (2017). Multicenter analysis of impact of anastomotic leakage on long-term oncologic outcomes after curative resection of colon cancer. Surgery.

[54] Eriksen M.T., Wibe A., Norstein J., Haffner J., Wiig J.N. (2005). Anastomotic leakage following routine mesorectal excision for rectal cancer in a national cohort of patients. Colorectal Dis..

[55] Espin E., Ciga M.A., Pera M., Ortiz H. (2015). Oncological outcome following anastomotic leak in rectal surgery. Br. J. Surg..

[56] Mirnezami A., Mirnezami R., Chandrakumaran K., Sasapu K., Sagar P., Finan P. (2011). Increased local recurrence and reduced survival from colorectal cancer following anastomotic leak: Systematic review and meta-analysis. Ann. Surg..

[57] Lu Z.R., Rajendran N., Lynch A.C., Heriot A.G., Warrier S.K. (2016). Anastomotic Leaks After Restorative Resections for Rectal Cancer Compromise Cancer Outcomes and Survival. Dis. Colon Rectum.

[58] Ha G.W., Kim J.H., Lee M.R. (2017). Oncologic Impact of Anastomotic Leakage Following Colorectal Cancer Surgery: A Systematic Review and Meta-Analysis. Ann. Surg. Oncol..

[59] Furnée E.J.B., Aukema T.S., Oosterling S.J., Borstlap W.A.A., Bemelman W.A., Tanis P.J., Dutch Snapshot Research G. (2019). Influence of Conversion and Anastomotic Leakage on Survival in Rectal Cancer Surgery; Retrospective Cross-sectional Study. J. Gastrointest. Surg..

[60] Schietroma M., Pessia B., Carlei F., Cecilia E.M., De Santis G., Amicucci G. (2015). Laparoscopic versus open colorectal surgery for colon cancer: The effect of surgical trauma on the bacterial translocation. A prospective randomized study. Am. J. Surg..

[61] Grewal S., Korthouwer R., Bögels M., Braster R., Heemskerk N., Budding A.E., Pouw S.M., van Horssen J., Ankersmit M., Meijerink J. (2018). Spillage of bacterial products during colon surgery increases the risk of liver metastases development in a rat colon carcinoma model. OncoImmunology.

[62] Chin K.F., Kallam R., O’Boyle C., MacFie J. (2007). Bacterial translocation may influence the long-term survival in colorectal cancer patients. Dis. Colon Rectum.

[63] Koratzanis G., Giamarellos-Bourboulis E.J., Papalambros E., Giamarellou H. (2002). Bacterial translocation following intrabdominal surgery. Any influence of antimicrobial prophylaxis?. Int. J. Antimicrob. Agents.

[64] Reddy B.S., MacFie J., Gatt M., Macfarlane-Smith L., Bitzopoulou K., Snelling A.M. (2007). Commensal bacteria do translocate across the intestinal barrier in surgical patients. Clin. Nutr..

[65] Buttenschoen K., Buttenschoen D.C., Berger D., Vasilescu C., Schafheutle S., Goeltenboth B., Seidelmann M., Beger H.G. (2001). Endotoxemia and acute-phase proteins in major abdominal surgery. Am. J. Surg..

[66] Buttenschoen K., Schneider M.E., Utz K., Kornmann M., Beger H.G., Buttenschoen D.C. (2009). Effect of major abdominal surgery on endotoxin release and expression of Toll-like receptors 2/4. Langenbeck’s Arch. Surg. Dtsch. Ges. Chir..

[67] Schietroma M., Carlei F., Cappelli S., Amicucci G. (2006). Intestinal permeability and systemic endotoxemia after laparotomic or laparoscopic cholecystectomy. Ann. Surg..

[68] Rosadini C.V., Kagan J.C. (2017). Early innate immune responses to bacterial LPS. Curr. Opin. Immunol..

[69] Kawai T., Akira S. (2010). The role of pattern-recognition receptors in innate immunity: Update on Toll-like receptors. Nat. Immunol..

[70] El Kasmi K.C., Anderson A.L., Devereaux M.W., Fillon S.A., Harris J.K., Lovell M.A., Finegold M.J., Sokol R.J. (2012). Toll-like receptor 4-dependent Kupffer cell activation and liver injury in a novel mouse model of parenteral nutrition and intestinal injury. Hepatology.

[71] Leong H.S., Robertson A.E., Stoletov K., Leith S.J., Chin C.A., Chien A.E., Hague M.N., Ablack A., Carmine-Simmen K., McPherson V.A. (2014). Invadopodia are required for cancer cell extravasation and are a therapeutic target for metastasis. Cell Rep..

[72] Chen D.S., Mellman I. (2017). Elements of cancer immunity and the cancer-immune set point. Nature.

[73] Galon J., Costes A., Sanchez-Cabo F., Kirilovsky A., Mlecnik B., Lagorce-Pagès C., Tosolini M., Camus M., Berger A., Wind P. (2006). Type, density, and location of immune cells within human colorectal tumors predict clinical outcome. Science.

[74] Cavnar M.J., Turcotte S., Katz S.C., Kuk D., Gonen M., Shia J., Allen P.J., Balachandran V.P., D’Angelica M.I., Kingham T.P. (2017). Tumor-Associated Macrophage Infiltration in Colorectal Cancer Liver Metastases is Associated With Better Outcome. Ann. Surg. Oncol..

[75] Tanis E., Julie C., Emile J.F., Mauer M., Nordlinger B., Aust D., Roth A., Lutz M.P., Gruenberger T., Wrba F. (2015). Prognostic impact of immune response in resectable colorectal liver metastases treated by surgery alone or surgery with perioperative FOLFOX in the randomised EORTC study 40983. Eur. J. Cancer.

[76] Meshcheryakova A., Tamandl D., Bajna E., Stift J., Mittlboeck M., Svoboda M., Heiden D., Stremitzer S., Jensen-Jarolim E., Grunberger T. (2014). B cells and ectopic follicular structures: Novel players in anti-tumor programming with prognostic power for patients with metastatic colorectal cancer. PLoS ONE.

[77] Yang Z., Zhang M., Peng R., Liu J., Wang F., Li Y., Zhao Q., Liu J. (2020). The prognostic and clinicopathological value of tumor-associated macrophages in patients with colorectal cancer: A systematic review and meta-analysis. Int. J. Colorectal Dis..

[78] Zhang F., Wang H., Wang X., Jiang G., Liu H., Zhang G., Wang H., Fang R., Bu X., Cai S. (2016). TGF-beta induces M2-like macrophage polarization via SNAIL-mediated suppression of a pro-inflammatory phenotype. Oncotarget.

[79] Fridlender Z.G., Sun J., Kim S., Kapoor V., Cheng G., Ling L., Worthen G.S., Albelda S.M. (2009). Polarization of tumor-associated neutrophil phenotype by TGF-beta: “N1” versus “N2” TAN. Cancer Cell.

[80] Norton S.E., Dunn E.T., McCall J.L., Munro F., Kemp R.A. (2016). Gut macrophage phenotype is dependent on the tumor microenvironment in colorectal cancer. Clin. Transl. Immunol..

[81] Edin S., Wikberg M.L., Oldenborg P.A., Palmqvist R. (2013). Macrophages: Good guys in colorectal cancer. Oncoimmunology.

[82] Matsubara D., Arita T., Nakanishi M., Kuriu Y., Murayama Y., Kudou M., Konishi H., Komatsu S., Shiozaki A., Otsuji E. (2020). The impact of postoperative inflammation on recurrence in patients with colorectal cancer. Int. J. Clin. Oncol..

[83] Kuraishy A., Karin M., Grivennikov S.I. (2011). Tumor promotion via injury- and death-induced inflammation. Immunity.

[84] Kimura F., Shimizu H., Yoshidome H., Ohtsuka M., Miyazaki M. (2010). Immunosuppression following surgical and traumatic injury. Surg. Today.

[85] Shankaran V., Ikeda H., Bruce A.T., White J.M., Swanson P.E., Old L.J., Schreiber R.D. (2001). IFNgamma and lymphocytes prevent primary tumour development and shape tumour immunogenicity. Nature.

[86] Huang Y.-M., Lee Y.-W., Huang Y.-J., Wei P.-L. (2020). Comparison of clinical outcomes between laparoscopic and open surgery for left-sided colon cancer: A nationwide population-based study. Sci. Rep..

[87] Lacy A.M., Garcia-Valdecasas J.C., Delgado S., Castells A., Taura P., Pique J.M., Visa J. (2002). Laparoscopy-assisted colectomy versus open colectomy for treatment of non-metastatic colon cancer: A randomised trial. Lancet.

[88] Bonjer H.J., Deijen C.L., Abis G.A., Cuesta M.A., van der Pas M.H., de Lange-de Klerk E.S., Lacy A.M., Bemelman W.A., Andersson J., Angenete E. (2015). A randomized trial of laparoscopic versus open surgery for rectal cancer. N. Engl. J. Med..

[89] Jayne D.G., Guillou P.J., Thorpe H., Quirke P., Copeland J., Smith A.M., Heath R.M., Brown J.M. (2007). Randomized trial of laparoscopic-assisted resection of colorectal carcinoma: 3-year results of the UK MRC CLASICC Trial Group. J. Clin. Oncol..

[90] Buunen M., Veldkamp R., Hop W.C., Kuhry E., Jeekel J., Haglind E., Pahlman L., Cuesta M.A., Msika S., Morino M. (2009). Survival after laparoscopic surgery versus open surgery for colon cancer: Long-term outcome of a randomised clinical trial. Lancet Oncol..

[91] Jayne D.G., Thorpe H.C., Copeland J., Quirke P., Brown J.M., Guillou P.J. (2010). Five-year follow-up of the Medical Research Council CLASICC trial of laparoscopically assisted versus open surgery for colorectal cancer. Br. J. Surg..

[92] Khatib A.M., Fallavollita L., Wancewicz E.V., Monia B.P., Brodt P. (2002). Inhibition of hepatic endothelial E-selectin expression by C-raf antisense oligonucleotides blocks colorectal carcinoma liver metastasis. Cancer Res..

[93] Vidal-Vanaclocha F., Fantuzzi G., Mendoza L., Fuentes A.M., Anasagasti M.J., Martin J., Carrascal T., Walsh P., Reznikov L.L., Kim S.H. (2000). IL-18 regulates IL-1beta-dependent hepatic melanoma metastasis via vascular cell adhesion molecule-1. Proc. Natl. Acad. Sci. USA.

[94] Grose R., Hutter C., Bloch W., Thorey I., Watt F.M., Fassler R., Brakebusch C., Werner S. (2002). A crucial role of beta 1 integrins for keratinocyte migration in vitro and during cutaneous wound repair. Development.

[95] Watt F.M., Fujiwara H. (2011). Cell-extracellular matrix interactions in normal and diseased skin. Cold Spring Harb. Perspect. Biol..

[96] Ramphal W., Boeding J.R.E., Gobardhan P.D., Rutten H.J.T., de Winter L., Crolla R., Schreinemakers J.M.J. (2018). Oncologic outcome and recurrence rate following anastomotic leakage after curative resection for colorectal cancer. Surg. Oncol..

[97] Noh G.T., Ann Y.S., Cheong C., Han J., Cho M.S., Hur H., Min B.S., Lee K.Y., Kim N.K. (2016). Impact of anastomotic leakage on long-term oncologic outcome and its related factors in rectal cancer. Medicine.

[98] Wright H.L., Moots R.J., Bucknall R.C., Edwards S.W. (2010). Neutrophil function in inflammation and inflammatory diseases. Rheumatology.

[99] Fang F.C. (2004). Antimicrobial reactive oxygen and nitrogen species: Concepts and controversies. Nat. Rev. Microbiol..

[100] Horowitz M., Neeman E., Sharon E., Ben-Eliyahu S. (2015). Exploiting the critical perioperative period to improve long-term cancer outcomes. Nat. Rev. Clin. Oncol..

[101] Neeman E., Zmora O., Ben-Eliyahu S. (2012). A new approach to reducing postsurgical cancer recurrence: Perioperative targeting of catecholamines and prostaglandins. Clin. Cancer Res..

[102] Alverdy J.C., Hyoju S.K., Weigerinck M., Gilbert J.A. (2017). The gut microbiome and the mechanism of surgical infection. Br. J. Surg..

[103] Donaldson G.P., Lee S.M., Mazmanian S.K. (2016). Gut biogeography of the bacterial microbiota. Nat. Rev. Microbiol..

[104] Gessler B., Eriksson O., Angenete E. (2017). Diagnosis, treatment, and consequences of anastomotic leakage in colorectal surgery. Int. J. Colorectal Dis..

[105] Markar S., Gronnier C., Duhamel A., Mabrut J.Y., Bail J.P., Carrere N., Lefevre J.H., Brigand C., Vaillant J.C., Adham M. (2015). The Impact of Severe Anastomotic Leak on Long-term Survival and Cancer Recurrence After Surgical Resection for Esophageal Malignancy. Ann. Surg..

[106] Zandstra D.F., Van Saene H.K. (2011). Selective decontamination of the digestive tract as infection prevention in the critically ill. A level 1 evidence-based strategy. Minerva Anestesiol..

[107] Farran L., Llop J., Sans M., Kreisler E., Miro M., Galan M., Rafecas A. (2008). Efficacy of enteral decontamination in the prevention of anastomotic dehiscence and pulmonary infection in esophagogastric surgery. Dis. Esophagus.

[108] Roos D., Dijksman L.M., Sondermeijer B.M., Oudemans-van Straaten H.M., De Wit L.T., Gerhards M.F. (2009). Perioperative selective decontamination of the digestive tract (SDD) in elective colorectal surgery. J. Gastrointest. Surg..

[109] Roos D., Dijksman L.M., Oudemans-van Straaten H.M., de Wit L.T., Gouma D.J., Gerhards M.F. (2011). Randomized clinical trial of perioperative selective decontamination of the digestive tract versus placebo in elective gastrointestinal surgery. Br. J. Surg..

